# The Efficacy of Bevacizumab Compared with Other Targeted Drugs for Patients with Advanced NSCLC: A Meta-Analysis from 30 Randomized Controlled Clinical Trials

**DOI:** 10.1371/journal.pone.0062038

**Published:** 2013-04-16

**Authors:** Jianlan Cui, Xueya Cai, Min Zhu, Tianshu Liu, Naiqing Zhao

**Affiliations:** 1 Department of Biostatistics, Fudan University, Shanghai, People's Republic of China; 2 Department of Biostatistics and Computational Biology, University of Rochester School of Medicine and Dentistry, Rochester, New York, United States of America; 3 AIDS and TB Division, Shanghai Zhabei Center for Disease Control and Prevention, Shanghai, People's Republic of China; 4 Department of Medical Oncology, Zhongshang Hospital, Fudan University, Shanghai, People's Republic of China; 5 Key Laboratory of Public Health Safety, Fudan University, Ministry of Education, Shanghai, People's Republic of China; Kyushu University, Japan

## Abstract

**Background:**

The extent of the benefit of bevacizumab combined with chemotherapy in the treatment of advanced non-small-cell lung cancer (NSCLC) is still unclear. We performed this meta-analysis to compare the efficacy of bevacizumab with other commonly used targeted drugs for different patients with advanced NSCLC.

**Methods:**

We searched PubMed, Cochrane Library, EMBASE and abstracts from the proceedings of the American Society of Clinical Oncology (ASCO), and identified 30 randomized controlled clinical trials published within 1999 to 2011 for meta-analysis.

**Results:**

The outcomes of treatment efficacy included response rate, PFS and OS. Comparing bevacizumab (15 mg/kg) with chemotherapy to standard chemotherapy alone, for chemotherapy-naïve patients, the pooled OR of response rate was 2.741(95%CI: 2.046, 3.672), the pooled HR for disease progression was 0.645 (95%CI: 0.561, 0.743), and the pooled HR for death was 0.790 (95%CI: 0.674, 0.926), respectively. In addition, the adjusted HR for previously-treated patients was 0.680 (95%CI: 0.492, 0.942) comparing bevacizumab combined with chemotherapy to standard chemotherapy alone.

**Conclusions:**

Bevacizumab accompanied by chemotherapy was found to significantly improve patients' response rate, progression free survival (PFS), and overall survival (OS) among chemotherapy-naïve patients compared to other targeted drugs in the treatment of non-small cell lung carcinoma (NSCLC).

## Introduction

Lung cancer has become the most common cancer and the leading cause of cancer death in the world [1], [2]. Non-small cell lung cancer accounts for at least 85% in all lung cancer cases [3], presenting as local advanced disease in approximately 25–30% of cases and as metastatic disease in approximately 40–50% of cases [4]. Various epidemiological studies have shown that the 5-year survival rate for patients with NSCLC is extremely low, ranging from 5% to 15% [2]. For NSCLC patients with local advanced or metastatic disease, chemotherapy, radiation and supportive treatment are the principal therapies given the fact that these patients are not able to tolerate surgical operations. However, standard first-line chemotherapy has limited efficacy for NSCLC patients, with an objective response rate about 30%, median survival time 8–9 months and 1-year survival rate 30–40% [5], all of which call for a more effective and safer therapy for lung cancer.

In general, aberrant biological pathways in tumorigenesis result in the disfunction of a protein molecule or a gene fragment, mostly at the molecular level. Accordingly, recent clinical trials have focused on targeted therapies designed to interfere with specific aberrant biological pathways as a new treatment option for NSCLC [6]. Studies, including a recent meta-analysis report, have showed that the use of chemotherapy plus Bevacizumab (at a dose of 15 mg/kg, every 3 weeks) increases two year survival rate for patients diagnosed with advanced lung cancer compared to chemotherapy alone[7], [8]. The main agents that have been investigated so far in NSCLC treatment are epidermal growth factor receptor (EGFR) family (tyrosine kinase) inhibitors (gefitinib and erlotinib), monoclonal antibodies targeting EGFR (cetuximab), and anti-VEGF monoclonal antibody (bevacizumab).

In different clinical trials, the hazard ratios for PFS and OS of bevacizumab use ranged from 0.55 to 0.85 and from 0.71 to 1.03, respectively [9]–[14]. In terms of gefitinib use, the ranges of hazard ratios for PFS and OS were from 0.30 to 1.09 and from 0.77 to 1.64, respectively [15]–[17], which overlapped those of bevacizumab. Similarly, controversial and inefficient results have been reported for other targeted drugs in studies with small sample size and/or different inclusion and exclusion criteria.

In this study we performed an updated meta-analysis to systematically study the efficacy of bevacizumab combined with chemotherapy for advanced NSCLC patients. Our meta-analysis is different from the previous ones in that we target to provide information for future research in comparisons between bevacizmab and other targeted drugs. Information used in the study was obtained from reported and unreported randomized controlled clinical trial studies, and targeted drugs included gefitinib, erlotinib and cetuximab. Our meta-analysis has a higher power in testing efficacy compared to previously reported individual clinical trials, and will help make evidence-based clinical decisions for the treatment of NSCLC.

## Materials and Methods

### 1. Searching method

An electronic search of the PubMed database, the Cochrane Library, and the EMBASE was performed, with the keywords ((non-small-cell lung cancer) OR nsclc) AND (target* therapy). The published language was limited to English and the years were limited from 1999 to 2011. MeSH terms searching was performed in PubMed. The American Society of Clinical Oncology (ASCO) Annual Meeting abstracts were also searched from 2000 to 2011. At the same time, the reference of related systematic reviews and clinical trials were screened.

### 2. Inclusion Criteria

The relevant clinical trials were manually selected carefully based on the following criteria: (1) randomized controlled trial (RCT); (2) patients with confirmed stage IIIB, stage IV or recurrent NSCLC based on historical or cytological evidence; (3) placebo-controlled or other types of superiority trial as well as non-inferiority trial; (4) Information collected including response rate, hazard ratio for progression free survival and overall survival, along with their 95% CIs or relevant data.

When searched references referred to same studies, the most recently published papers were chosen.

### 3. Efficacy indicators

Objective response rate (ORR) is defined as the proportion of complete response (CR) plus partial response (PR) among evaluable patients. Progression free survival (PFS) is defined as the duration of time from random assignment to documented disease progression or death, whichever occurs first. Overall survival (OS) is defined as the time from random assignment to death, irrespective of the cause of death. For patients with no event observed, the time to censor refers to the time to last follow-up. The treatment efficacy of targeted drug compared to alternative drugs was measured by odds ratio for response rate (OR_ORR_), and hazard ratio for progression free survival and overall survival (HR_PFS_ or HR_OS_).

### 4. Quality assessment

The methodological quality of trials was evaluated using the Jadad scale [a 5-point scale assessing randomization (0–2 points), double-blinding (0–2 points), and follow-up (0–1 points)] [18]. The Jadad scale has a total range from 0 to 5, and clinical trials are defined as ‘good’ when the scale is 3–5 [18]. Two reviewers independently assessed trial quality, and disagreements were resolved by consensus.

### 5. Data extraction

Two investigators searched the publications independently using standardized data-abstraction forms. When the two investigators discovered different results, an independent expert in oncology made the final decision of study conclusions. Information collected from these publications included first author, year of publication, targeted treatment, chemotherapy regimens, number of centers, number of patients, patient characteristics, study design (blinded or not), and the outcomes. Outcomes collected from these studies included response rate, median PFS and OS, hazard ratios for PFS and OS (HR_PFS_ or HR_OS_) and their 95% confidence intervals (CIs), and adverse events. In addition, patient characteristics collected from these studies included median age, the percentage of female, percentage of stage IV patients, ECOG performance status, and whether EGFR expression as entry criteria,

When HRs were not reported in collected papers, we computed HRs and its confidence intervals assuming an exponential distribution of the survival curve. In the estimation of HRs, we applied the published methodology [19] on the graphic software package Engauge to estimate the logarithm transformed HR and variance from the Kaplan–Meier curves.

### 6. Statistical analysis

Analyses were performed in intention-to-treat (ITT) population. We first tested the statistical heterogeneity between trials (meaningful differences between studies) using the chi-squared Q-test based on the fixed-effect model. The clinical trials were considered heterogeneous when the P value of the chi-squared Q-test was less than 0.10, or when I^2^ was greater than 50%. When the analyses showed heterogeneity between different clinical trials, a random effect model was applied to accommodate the heterogeneity [20]. The pooled odds ratios for response rate (OR_ORR_), HRs for PFS and OS (HR_PFS_ or HR_OS_) were calculated. We decided to present three primary measures to show the treatment effect from different angles because PFS and OS can better describe the efficacy of a targeted drug than response rate. In addition, it is not uncommon to detect discrepancy between a clear benefit in PFS and a vague benefit in OS for lung cancer patients [21]–[23]. Furthermore, we estimated and tested the difference of treatment effect between bevacizumab combined with chemotherapy and other targeted drugs using the meta-regression model. The crude and risk-adjusted 95% confidence interval were reported when the models included/excluded patient characteristics. To demonstrate whether the progression free survival was associated with stable disease (SD) or objective response rate (ORR) to the medication, or both, we performed the additional analysis of logarithm transformed outcomes (HR_PFS_) against use of bevacizumab and OR_ORR_, controlling for patient characteristics (median age, mean ECOG performance score) and study design (chemotherapy type for the control group). Similarly, logarithm transformed HR_OS_ was modeled against HR_PFS_ and bevacizumab.

In addition to the above tests, we performed imputation study to test the influence of each individual study using the leave-one-out strategy [20]. Finally, we performed the funnel plot as well as Begg's and Egger's tests to examine potential publication bias.

We performed subgroup analysis in this study based on patient treatment status using the meta-regression models. Chemotherapy-naïve patients were defined as those with no prior chemotherapy and no previous treatment with EGFR-targeted drugs or monoclonal antibodies. Previously-treated patients were defined as patients progressed or recurred after at least one previous chemotherapy regimen.

All the analyses were performed using STATA 11.0.

The study was written according to the Preferred Reporting Items for Systematic Reviews and Meta-Analyses (PRISMA) statement [24].

## Results

**Figure 1:**
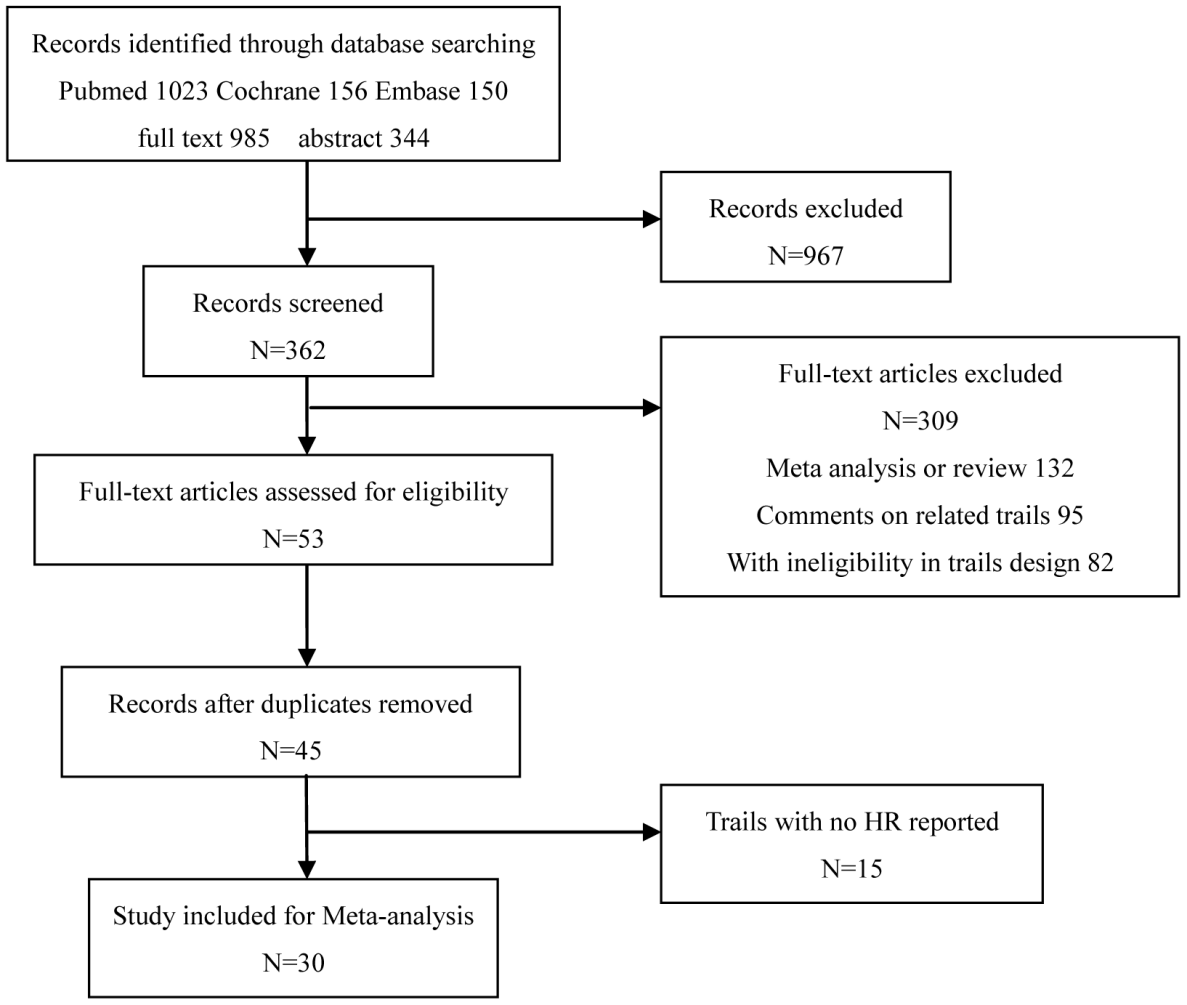
Flow chart showing the progress of trials through the review.

The flowchart of our study is shown in Figure1. From 1,329 published papers and abstract that we found, 967 were excluded from this study based on our inclusion/exclusion criteria. In addition, 309 articles were further excluded if they were already review papers or comments. Among the 53 articles that were left from the above exclusion criteria, five articles were excluded since they were duplicate reports. Finally, 15 additional articles were excluded since they did not report outcomes relevant to our study. Our final sample included 15,650 patients collected from 30 randomized clinical trials.

**Figure 2:**
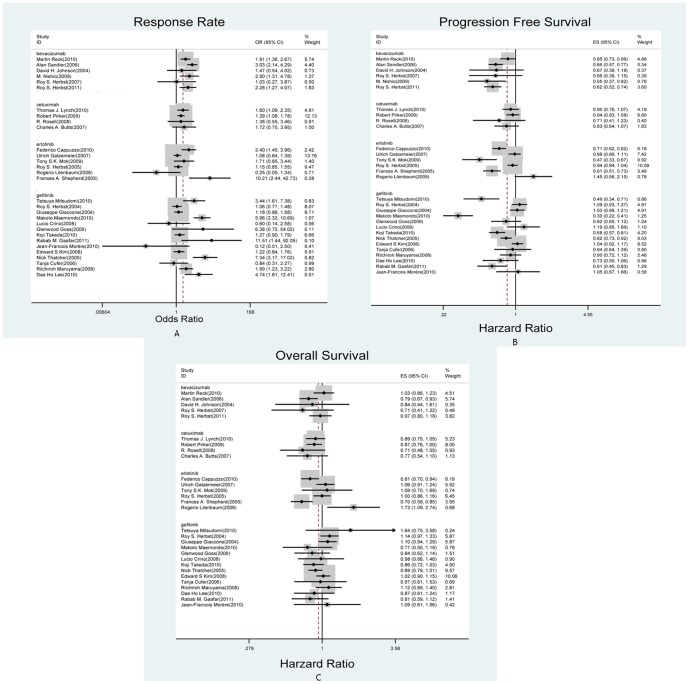
Forest plots of individual trials. A: Odds ratio of response rate; B: Hazard ratio of progression free survival; C: Hazard ratio of overall survival.

Among the 30 multi-center randomized clinical trials [9]–[17], [25]–[45] we included in this study, 13 were double-blinded trials. All of these studies were published in peer-reviewed journals except one that published as an abstract in ASCO annual meeting. Six of the clinical trials applied bevacizumab 15 mg/kg every 3 weeks combined with targeted treatment, four of them applied cetuximab (400 mg/m2, initial dose followed by 250 mg/m2 every week), six of them applied erlotinib 150 mg/d, and the other fourteen clinical trials applied gefitinib 250 mg/d (Table 1). The patient level analyses showed that patient median age varied from 58 to 71, percent of female varied from 12% to 69.8%, and 65–100% of patients having cancer stage higher than 3 in different trials. Individual results of included trials were summarized in figure 2.

**Table 1:** Baseline characteristics of the thirty trials.

First Author	No. of centers	Jadad Score			EGFR mutation	CT- naive	Asian origin	Group	n	Median age	Female (%)	ECOG> = 2(%)	Stage > = IV(%)
		random	blind	dropouts									
Reck M. (2010)	150	1	2	1	No	Yes	No	GP+bev	351	59	37.6	0	84
								GP	347	59	35.7	0	83.9
Sandler A.(2006)	NR	1	0	1	No	Yes	No	PCp +bev	417	NR	50	0	88
								PCp	433	NR	42	0	87
Johnson DH. (2004)	12	2	1	1	No	No	No	PCp +bev	35	NR	54.28*	11.43	80
								PCp	32	NR	25	6.25	81.25
Nishio M. (2009)	NR	1	0	1	No	No	Yes	PCp +bev	121	NR	NR	NR	NR
								PCp	59	NR	NR	NR	NR
Herbst RS. (2007)	51	1	0	1	No	Yes	No	D/M +bev	40	63.5	42.5	0	NR
								D/M	41	65	39	2.4	NR
Herbst RS. (2011)	177	2	2	1	No	Yes	No	erl+bev	319	64.8	46	7	NR
								erl	317	65	46	6	NR
Lynch TJ. (2010)	96	1	0	1	No	Yes	No	TC+cet	325	64	43	0	93
								TC	320	65	40	0	90
Pirker R. (2009)	155	2	0	1	Yes	Yes	No	NP+cet	557	59	31	17	94
								NP	568	60	29	18	94
Rosell R. (2008)	16	1	0	0	Yes	Yes	No	NP+cet	43	58	23.3	93	93
								NP	43	57	27.9	93	88
Butts CA. (2007)	32	1	0	1	No	Yes	No	GP+cet	65	66	61.5*	1.5	84.6
								GP	66	64	50	1.5	83.3
Cappuzzo F. (2010)	110	2	1	1	No	Yes	No	erl	438	60	27	0	74
								placebo	451	60	25	0	76
Gatzemeier U. (2007)	164	1	1	1	No	Yes	No	GP+erl	580	61	22	<1	65
								GP	579	60	25	<1	67
Mok T. (2009)	19	2	1	1	No	Yes	Yes	GP+erl	76	57.5	29	0	83
								GP	78	57.0	31	2.4	79
Herbst RS. (2005)	multi	1	1	1	No	No	No	PCp+erl	539	63	40.3	0	84.4
								PCp	540	63	38.4	0.2	82.2
Lilenbaum R. (2008)	14	1	0	1	No	Yes	No	erl	52	NR	56*	NR	87
								PCp	51	NR	45	NR	86
Shepherd FA. (2005)	82	2	1	1	No	Yes	No	erl	488	62	35.5	25.8	NR
								placebo	243	59	34.2	23.0	NR
Mitsudomi T. (2010)	36	2	0	1	Yes	Yes	Yes	gef	86	64.0	68.6	0	88.4
								Cisplatin +docetaxel	86	64.0	69.8	0	89.6
Herbst RS. (2004)	multi	1	1	0	No	Yes	No	PCp+gef	345	61	42.3	10.4	97.4
								PCp	345	63	38.6	9.3	95.4
Giaccone G. (2004)	155	1	1	0	No	Yes	No	GP+gef	365	59	23.3	9.6	98.1
								GP	363	61	27.8	9.6	97.0
Maemondo M. (2010)	43	1	0	1	Yes	Yes	Yes	gef	114	63.9	63.2	0.9	86.8
								PCp	114	62.6	64	1.8	81.6
Crino L. (2008)	41	1	0	1	No	Yes	No	gef	97	74	22.7	23.7*	NR
								vinorelbine	99	74	26.3	16.2	NR
Goss G. (2009)	37	1	2	1	No	Yes	No	gef	100	74	39.0	100	NR
								placebo	101	76	39.6	100	NR
Takeda K. (2010)	39	2	0	1	No	Yes	Yes	Platinum +gef	300	62	36*	0	81.7
								platinum	298	63	67.8	0	81.9
Gaafar RM. (2011)	24	2	1	1	No	No	No	gef	86	61	22	7	100
								placebo	87	62	24	5	100
Morère JF. (2010)	29	1	0	1	No	No	No	gef	43	70	12*	100	100
								docetaxel	42	71	21	100	100
Kim ES. (2008)	149	2	0	1	No	No	No	gef	733	61	36.4	11.7	77.9
								docetaxel	733	60	33.4	11.5	81.1
Thatcher N. (2005)	210	2	2	1	No	No	No	gef	1129	62	33	0	81
								placebo	563	61	33	0	80
Cufer T. (2006)	25	2	0	1	No	No	No	gef	68	63.0	31	36.8*	NR
								docetaxel	73	59.5	30	28.8	NR
Maruyama R. (2008)	50	1	0	1	No	Yes	Yes	gef	245	NR	38.4	4.5	80.8
								docetaxel	244	NR	38.1	4.1	79.5
Lee DH. (2010)	6	1	0	1	No	Yes	Yes	gef	82	57	32.9*	7.3	86.6
								docetaxel	79	58	43.0	6.3	82.3

NR: not reported.

* unbalanced between groups.

CT: chemotherapy; bev: bevacizumab; erl: erlotinib; cet: cetuximab; gef: gefitinib.

GP: Cisplatin-Gemcitabine; PCp: Paclitaxel-carboplatin; TC: Taxane-carboplatin; NP: cisplatin-vinorelbine; D/M:docetaxel/pemetrexed.

**Figure 3:**
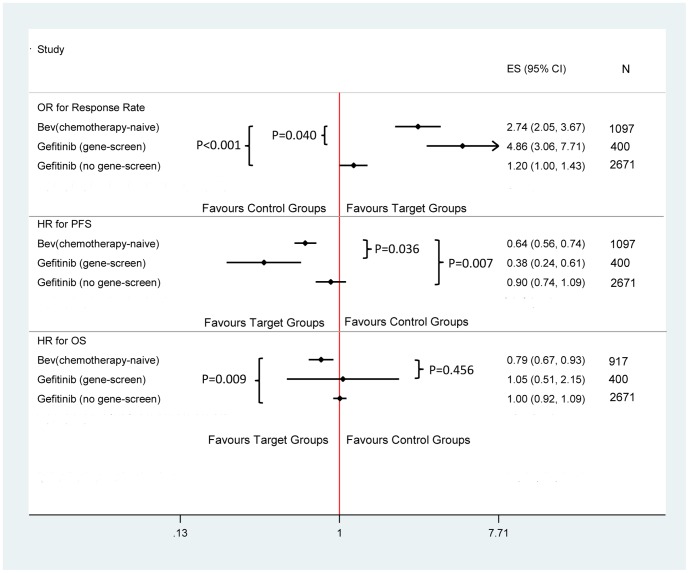
Response rate, PFS, OS of Bevacizumab versus Gefitinib in NSCLC patients with different EGFR status.

Among the 30 clinical trials included in the meta-analysis, 25 reported hazard ratios for PFS and OS (HR_PFS_ and HR_OS_) and the corresponding 95% confidence intervals (CIs). For other 5 trials, 3 reported the HR_PFS_ directly and 2 reported the HR_OS_ directly. In terms of the efficacy for patients treated with gefitinib (2 trials [15], [17] for EGFR-mutated patients among 14 clinical trials), meta-analysis showed that pooled OR_ORR_ in EGFR-mutated patients was 4.862 (95%CI: 3.064, 7.715; I^2^ = 20.2%; Figure 3) compared to 1.199 (95%CI: 1.003, 1.434; I^2^ = 43.3%) in EGFR untested patients (P<0.001). Pooled HR_PFS_ in EGFR-mutated patients (0.379, 95%CI: 0.235, 0.611; I^2^ = 74.2%) was smaller than that in EGFR untested patients (0.896, 95%CI: 0.738, 1.087; I^2^ = 79.1%, P = 0.001). In addition, pooled HR_OS_ in EGFR-mutated patients was 1.046 (95%CI: 0.509, 2.149; I^2^ = 63.0%), compared to 1.005 (95%CI: 0.924, 1.093; I^2^ = 38.5%) in EGFR untested patients (P = 0.914). Therefore, in the following comparison, we compared bevacizumab with other targeted drugs (gefitinib, erlotinib and cetuximab) in EGFR untested patients. However, in terms of HR_OS_, the comparison was made in both EGFR-mutated and EGFR untested patients.

**Figure 4:**
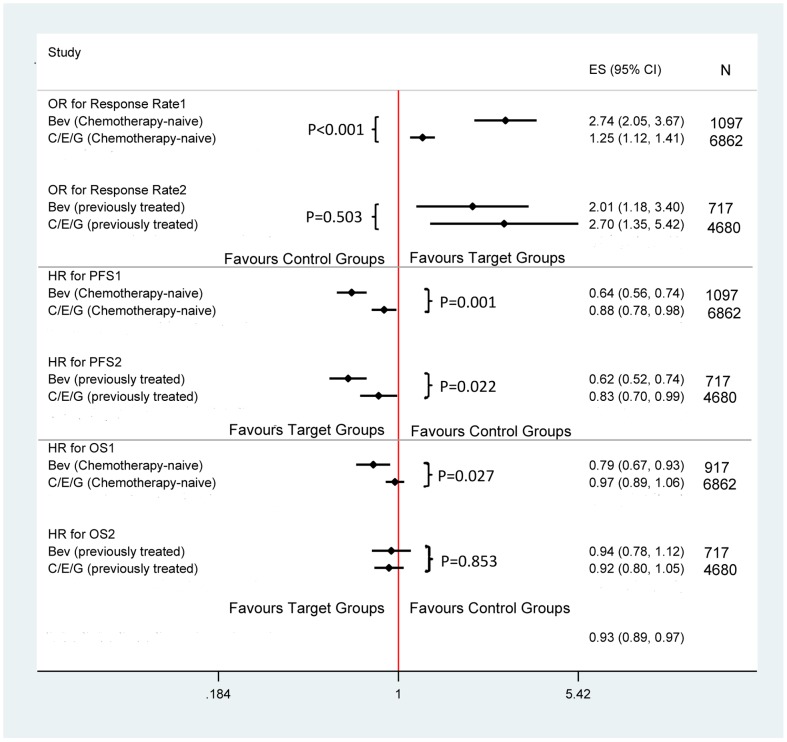
Response rate, PFS, OS of Bevacizumab versus other targeted drugs in EGFR untested NSCLC patients.

In terms of efficacy for chemotherapy-naïve patients, a higher pooled OR_ORR_ was found in trials applying bevacizumab (2.741, 95%CI: 2.046, 3.672; I2 = 0.0%) than those applying other targeted drugs (OR = 1.255, 95%CI: 1.117, 1.410; I^2^ = 48.9%) for chemotherapy-naïve patients (P<0.001, Figure 4). The pooled HR_PFS_ was found to be lower in trials applying bevacizumab (HR = 0.645, 95%CI: 0.561, 0.743; I^2^ = 0.0%) than those applying other targeted drugs (HR = 0.875, 95%CI: 0.779, 0.982; I^2^ = 78.5%, P = 0.001). In addition, the pooled HR_OS_ was found to be lower in trials applying bevacizumab (HR = 0.790, 95%CI: 0.674, 0.926; I^2^ = 0.0%) than those applying other targeted drugs (HR = 0.969, 95%CI: 0.889, 1.057; I^2 = ^50.2%, P = 0.027). Analysis for previously-treated patients showed that pooled OR_ORR_, HR_PFS_, and HR_OS_ were similar in trials applying bevacizumab versus other targeted drugs. For example, the OR_ORR_ was 2.008 (95%CI: 1.184, 3.404; I^2^ = 13.8%) and 2.704 (95%CI: 1.349, 5.424; I^2^ = 82.4%) for the two groups, respectively (P = 0.503); pooled HR_PFS_ was 0.624 (95%CI: 0.524, 0.742; I^2^ = 0.0%) and 0.831 (95%CI: 0.698, 0.989; I^2^ = 79.7%), respectively (P = 0.022). And the pooled HR_OS_ was 0.936 (95%CI: 0.780, 1.124; I^2^ = 11.6%) and 0.916(95%CI: 0.799, 1.051; I^2^ = 64.3%), respectively (P = 0.853).

**Figure 5:**
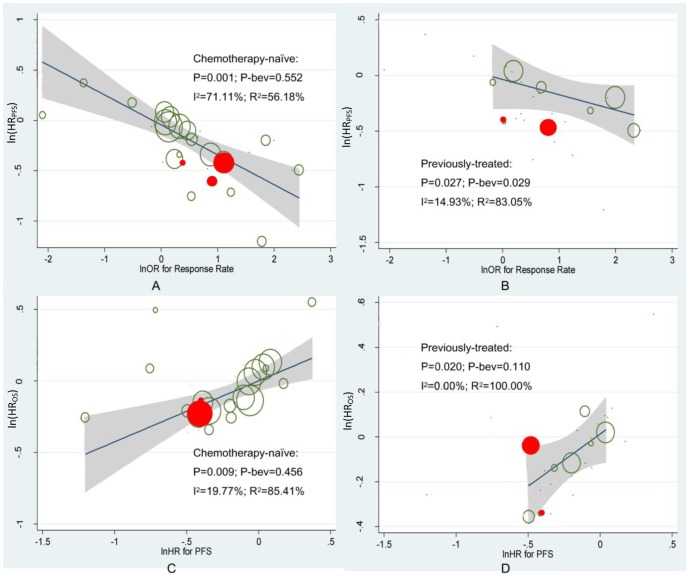
Results of meta-regression. A: ln(HR_PFS_) – ln(OR_ORR_), in chemotherapy-naïve patients; B: ln(HR_PFS_) – ln(OR_ORR_), in previously-treated patients; C: ln(HR_OS_) – ln(HR_PFS_), in chemotherapy-naïve patients; D: ln(HR_OS_) – ln(HR_PFS_), in previously-treated patients.

In chemotherapy-naïve patient, a meta-regression analysis showed that the overall lnHR_PFS_ was negatively associated with the lnOR_ORR_ (β = −0.251, P = 0.001; Figure 5 and Table 2). The subgroup analyses based on patient treatment status showed that the treatment of bevacizumab for previously-treatment patients was statistically different from those of other targeted drugs in terms of disease progression(P = 0.027). For HR_OS_, we found similar results for both chemotherapy-naïve patients and previously-treated patients (β = 0.374, P = 0.009; and β = 0.685, P = 0.020, Figure 5 and Table 2). Trials applying bevacizumab were marked in red and grey shaded areas with the confidence band for the regression line. The size of the circles represented the weight of each trial in the regression procedure.

**Table 2:** Crude and risk-adjusted hazard ratio of BEV comparing to C/E/G.

patients	Response variable	Treatment group	Number of trials	Crude		Adjusted	
				HR_Crude_	95%CI	HR_Adjusted_	95%CI
Chemotherapy-naïve	HR_PFS_	Bev	3	0.753	(0.570, 0.996)	0.847*	(0.687, 1.043)
		C/E/G	18	1	–	1	–
Previously-treated	HR_PFS_	Bev	2	0.758	(0.482, 1.191)	0.680*	(0.492,0.942)
		C/E/G	6	1	–	1	–
Chemotherapy-naïve	HR_OS_	Bev	2	0.774	(0.617, 0.972)	1.151**	(0.828, 1.600)
		C/E/G	18	1	–	1	–
Previously-treated	HR_OS_	Bev	2	0.985	(0.658, 1.475)	1.262**	(0.927, 1.710)
		C/E/G	6	1	–	1	–

*HR_adjusted_ was adjusted by ln(OR_ORR_).

** HR_adjusted_ was adjusted by ln(HR_PFS_).

**Figure 6:**
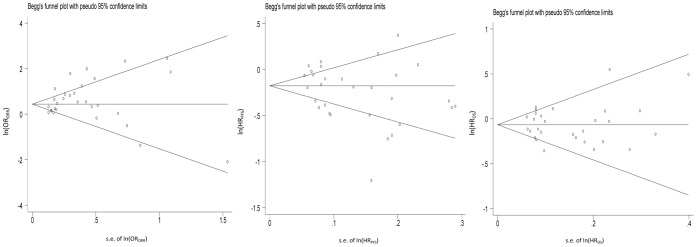
Begg's funnel plot.

The Begg's funnel tests were conducted to demonstrate the influence of publication bias (figure 6). The p-values were 0.301, 0.159 and 0.851, respectively.

## Discussion

Our meta-analyses showed that compared to other commonly used targeted drugs, chemotherapy with bevacizumab significantly improved patients' response rate, PFS and OS. The above findings were similar to previous findings [46]. In addition, bevacizumab provided significantly higher OR_ORR_, lower HR_PFS_, and lower HR_OS_ among chemotherapy-naïve patients, and lower HR_PFS_ among previous treated patients. It was also found that in EGFR-mutated patients, gefitinib significantly improved OR_ORR_ and reduces HR_PFS_. However, in general patients with EGFR status untested, bevacizumab showed a clear benefit in OR_ORR_, HR_PFS_, as well as HR_OS_, compared with gefitinib. These findings were consistent with previous publications [30].

Generally, mechanism of action of anticancer drugs was causing cancer cell death or blocking cancer cell growth. Objective response rate (ORR), which refers to the proportion of CR+PR, reflects the treatment effect by causing cancer cell death. On the other hand, SD reflects the treatment effect by blocking cancer cell growth. Our meta-regression models were performed to decompose the two treatment mechanisms among NSCLC patients by introducing ln(OR_ORR_) together with the bevacizumab indicator into the model. In these models we identified differences between the two types of targeted drugs in the contribution of blocking cell growth by estimating the adjusted bevacizumab effect, controlling the effect on contribution of killing tumor cells (OR_ORR_).

From the results (table 2), we found that in previously-treated patients, although bevacizumab was not outstanding in promoting beneficial events such as CR and PR, it surpassed other targeted drugs in maintaining the pharmacodynamic effect. This finding was consistent with the mechanism of bevacizumab which was slowing down the vessel growth instead of causing cell death. As we can see in figure 5, several trials with treatment group applying bevacizumab (marked in red) fall below the regression line, indicating that there are other factors contributing to the prolongation of PFS in spite of the elevation of ORR. The contribution of SD in PFS time is greater in the treatment group than in the control group.

We presented three primary measures (ORR, PFS and OS) to show the treatment effect of different targeted drugs. Response rate is greatly affected by the original volume of the solid tumor, average duration of administration, and the clinical stage of patients, while PFS and OS time can be greatly affected by the frequency of follow-up. These are possible reasons of having only one clinical trial (E4599) with significant overall survival benefit. Another possible reason of the negative findings in overall survival time may be the low power to detect significance due to small valid sample size. Simple meta-regression in this study showed significantly positive correlation between ln(HR_OS_) and ln(HR_PFS_) in both chemotherapy-naïve and previously treated patients, indicating that given a clear benefit in PFS, benefit in OS is much likely to be detected with a larger sample size (figure 5). In other words, we can eliminate the accelerated growth of tumor cells after disease progression which would result in a clear benefit in PFS but not in OS. Our finding that the crude but not the adjusted HR_OS_ of bevacizumab was significantly lower than that of other three drugs in chemotherapy-naïve patients indicated that the advantage in chemotherapy-naïve patients was mainly attributed to the elevation of ORR and prolongation of PFS. The finding that neither crude HR_OS_ nor adjusted HR_OS_ of bevacizumab was significantly different from those of other targeted drugs in previously treated patients may be explained by the complex and severity of patients.

Selection of target is essential in targeted therapies; therefore whether EGFR is mutated or not is of great significance in clinical decision. However, a considerable number of patients are unable to provide adequate tissue samples for accurate genotyping in practice. Our study showed that the benefit from bevacizumab was independent of EGFR status among a relatively large number of patients especially for those of first-line treatment. Such an effect was not able to detect for patients in second-line or third-line treatment, which suggests that patients may be more likely to show better response to the anti-angiogenic drug at early stage. Based on these findings, we would recommend early use of bevacizumab.

Limitations exist in this study. First, our meta-analysis cohort is heterogeneous regarding chemotherapies of the controls, and this may lead to unreliable findings. To address this issue, we performed an imputation study with leave-one-out strategy. The imputation analysis showed that the results had only slight difference when any single trial was removed from the meta-analysis, which indicates robustness of our study. Secondly, our analysis included a number of steps to minimize the potential for publication bias, including the Begg's test and Egger's test. The symmetrical distributions presented in Funnel plot showed a small number of outliers, which may result from the limit of published language. Third, with limited data information, our study was not able to control for heterogeneity of EGFR status in testing the treatment effect of different medications. However, literature shows that bevacizumab is an anti-VEGF mAb with a high affinity for VEGF [47]; therefore the treatment effect would not differ from the EGFR status of patients. In addition, when gefitinib was used, patients with EGFR mutated were found to have better treatment effects than those with unknown EGFR status (composed of both patients with EGFR mutation and those without EGFR mutation) [15], [34]. Given the fact that we found better treatment effect of bevacizumab comparing to gefitinib for patients with unkonwn EGFR status, we believe bevacizumab should show better treatment effect than gefitinib for patients without EGFR mutation.

Our study included clinical trials with only slightly different enrollment criteria and patient demographics. However patient characteristics (age, gender, ECOG performance status) were found not to be balanced between groups in a small number of trials. Such patient level difference may lead to heterogeneity in the meta-analysis. We carefully included aggregated patient characteristics into our meta regression level to control for heterogeneity in our study. Inconsistency of chemotherapies of the control group did exist in this analysis, which could not be eliminated due to the study background. Further analysis with Bayesian method might solve this problem [48].

Finally, the clinical trials collected in this study show high heterogeneity. Due to the relative small sample size, our analysis may not be considered as strong evidence of treatment effect as other meta-analysis although we controlled for patient characteristics as well as study design. A large RCT(s) or individual-patient data meta-analysis may be needed in the future to further examine the treatment difference.

In conclusion, we found from this meta-analysis study that for chemotherapy-naïve patients, the advantage of bevacizumab in HR_OS_ is mainly due to the elevation of ORR and prolongation of PFS. In addition, compared with other targeted drugs mentioned, chemotherapy with bevacizumab significantly improved patients' response rate, PFS and OS, especially for chemotherapy-naïve patients.

## Supporting Information

Table S1 **PRISMA Checklist.**
(DOC)
